# Combined Changes in Chloride Regulation and Neuronal Excitability Enable Primary Afferent Depolarization to Elicit Spiking without Compromising its Inhibitory Effects

**DOI:** 10.1371/journal.pcbi.1005215

**Published:** 2016-11-11

**Authors:** Petri Takkala, Yi Zhu, Steven A. Prescott

**Affiliations:** 1 Neurosciences and Mental Health, The Hospital for Sick Children, Toronto, Ontario, Canada; 2 Institute of Medical Science, University of Toronto, Toronto, Ontario, Canada; 3 Center for Pain Research, Department of Anesthesiology, University of Pittsburgh, Pittsburgh, Pennsylvania, United States of America; 4 Department of Physiology and the Institute of Biomaterials and Biomedical Engineering, University of Toronto, Ontario, Canada; George Mason University, UNITED STATES

## Abstract

The central terminals of primary afferent fibers experience depolarization upon activation of GABA_A_ receptors (GABA_A_R) because their intracellular chloride concentration is maintained above electrochemical equilibrium. Primary afferent depolarization (PAD) normally mediates inhibition via sodium channel inactivation and shunting but can evoke spikes under certain conditions. Antidromic (centrifugal) conduction of these spikes may contribute to neurogenic inflammation while orthodromic (centripetal) conduction could contribute to pain in the case of nociceptive fibers. PAD-induced spiking is assumed to override presynaptic inhibition. Using computer simulations and dynamic clamp experiments, we sought to identify which biophysical changes are required to enable PAD-induced spiking and whether those changes necessarily compromise PAD-mediated inhibition. According to computational modeling, a depolarizing shift in GABA reversal potential (*E*_GABA_) and increased intrinsic excitability (manifest as altered spike initiation properties) were necessary for PAD-induced spiking, whereas increased GABA_A_R conductance density (*ḡ*_GABA_) had mixed effects. We tested our predictions experimentally by using dynamic clamp to insert virtual GABA_A_R conductances with different *E*_GABA_ and kinetics into acutely dissociated dorsal root ganglion (DRG) neuron somata. Comparable experiments in central axon terminals are prohibitively difficult but the biophysical requirements for PAD-induced spiking are arguably similar in soma and axon. Neurons from naïve (i.e. uninjured) rats were compared before and after pharmacological manipulation of intrinsic excitability, and against neurons from nerve-injured rats. Experimental data confirmed that, in most neurons, both predicted changes were necessary to yield PAD-induced spiking. Importantly, such changes did not prevent PAD from inhibiting other spiking or from blocking spike propagation. In fact, since the high value of *ḡ*_GABA_ required for PAD-induced spiking still mediates strong inhibition, we conclude that PAD-induced spiking does not represent failure of presynaptic inhibition. Instead, diminished PAD caused by reduction of *ḡ*_GABA_ poses a greater risk to presynaptic inhibition and the sensory processing that relies upon it.

## Author Summary

Postsynaptic GABA_A_R mediate inhibition by causing hyperpolarization or by preventing (shunting) the depolarization caused by concurrent excitatory input. Presynaptic GABA_A_R work differently, in the spinal cord at least. Because of their higher-than-equilibrium intracellular chloride concentration, the central terminals of primary afferent fibers are depolarized by activation of GABA_A_R. This so-called primary afferent depolarization, or PAD, nonetheless reduces spike propagation and synaptic release from those fibers because of shunting effects and sodium channel inactivation. But those inhibitory effects can be diminished under certain pathological conditions; in fact, the emergence of dorsal root reflexes suggests that PAD can become paradoxically excitatory. The biophysical basis for this paradoxical excitation has been hinted at by experiments, but here, for the first time, we use computational modeling and dynamic clamp experiments to decipher how distinct contributing factors interact to enable PAD-induced spiking. Our results suggest that PAD-induced spiking requires a shift in GABA reversal potential plus changes in intrinsic excitability that allow for repetitive spiking during sustained depolarization. Inhibitory effects of PAD are retained under conditions in which GABA_A_R activation causes transient spiking and are only lost if GABA_A_R activation can evoke repetitive spiking.

## Introduction

Synaptic inhibition regulates transmission of sensory signals through the spinal cord. Importantly, numerous chronic pain conditions are associated with diminished inhibition [1–5] and pharmacological blockade of inhibition at the spinal level has been shown to reproduce many features of those chronic pain conditions [6–9]. Decreased transmitter release, reduced GABA_A_/glycine receptor function, and altered chloride regulation are all potential *dis*inhibitory mechanisms, but pre- and postsynaptic inhibition are not equally susceptible to certain pathological changes; for instance, the potassium-chloride co-transporter KCC2 is not expressed in primary afferent neurons, meaning disinhibitory effects of KCC2 downregulation [10] are attributable entirely to reduced postsynaptic inhibition, in cells that express KCC2. KCC3 is expressed in some primary afferents and can extrude chloride under isosmotic conditions [11,12] but it remains unknown whether KCC3 is altered under pathological conditions. Yet selective disruption of presynaptic inhibition can cause mechanical and thermal hypersensitivity [13] and presynaptic expression of the α2 GABA receptor subunit is necessary for the antihyperalgesic effect of diazepam [14]. These observations affirm that presynaptic GABA_A_R-mediated inhibition also plays a key role in nociception.

Pre- and postsynaptic inhibition in spinal cord are mechanistically distinct. Postsynaptically, in mature spinal neurons, the reversal potential associated with GABA_A_R (*E*_GABA_) is normally around -70 mV [10], meaning GABA_A_R activation reduces depolarization caused by concurrent excitatory input. Presynaptically, in the central terminals of primary afferents, *E*_GABA_ is normally around -35 mV because chloride is actively loaded into primary afferents by the sodium-potassium-chloride co-transporter NKCC1 [13,15–17], thus GABA_A_R activation causes depolarization. Contrary to the presumed excitatory effect of depolarization, primary afferent depolarization (PAD) mediates inhibitory effects via sodium channel inactivation and shunting [18–21]. However, PAD can sometimes trigger spikes that conduct antidromically, thus producing what is referred to as a dorsal root reflex (DRR) [22]. One theory holds that antidromically conducted spikes mediate an inhibitory effect by colliding with and blocking othrodromically conducted spikes originating in the periphery [23,24]; however, collisions are unlikely since the latency to travel the full length of the nerve is short relative to the interspike interval at realistic spiking rates. PAD-induced spikes are unlikely to trigger synaptic release from the PAD-affected branch because spike amplitude is attenuated, but PAD-induced spikes that manage to propagate to adjacent, PAD-free branches may trigger synaptic release [25]. The experiments required to test these model predictions are prohibitively difficult. The above theory was formulated for large myelinated proprioceptive afferents involved in locomotion; in contrast, within smaller afferents responsible for nociception, the prevailing view is that PAD-induced spikes occur only under pathological conditions and that DRRs contribute to neurogenic inflammation and hypersensitivity [22,26]. Within this context, PAD-induced spiking is thought to represent conversion of PAD from an inhibitory process to an excitatory one [22].

With respect to biophysical mechanisms, PAD-induced spiking requires GABA_A_R activation [27] and NKCC1-mediated chloride loading [28]. Enhanced chloride loading and the consequent depolarizing shift in *E*_GABA_ has been hypothesized to facilitate PAD-induced spiking [29,30]. Nerve injury increases NKCC1 protein levels and PAD [13,31], and although total NKCC1 expression is not altered by inflammation [32,33], NKCC1 membrane trafficking and phosphorylation are affected by painful stimuli [34]. Notably, inflammation causes a depolarizing shift in *E*_GABA_ [35] and promotes DRRs in C and Aδ fibers [36]. Increased GABA_A_R density and reduced low-threshold potassium channel density have also been hypothesized to promote DRRs [35,37] but the full set of requirements for PAD-induced spiking remains unclear. We sought to identify which biophysical changes, alone or together, enable PAD-induced spiking and how such changes impact PAD-mediated inhibition.

## Results

Changes in GABA conductance density *ḡ*_GABA_, its associated reversal potential *E*_GABA_, and intrinsic excitability have all been implicated in PAD-induced spiking, as outlined above. To account for whether a neuron spikes transiently or repetitively, and whether spike threshold is sensitive to the rate of depolarization, we discuss excitability in terms of spike initiation dynamics [38]. Rather than characterize further how excitability and GABAergic signalling are pathologically altered, we sought to decipher how known pathological alterations contribute to PAD-induced spiking. To this end, we took an approach distinct from previous studies to determine how isolated and combined changes in each factor–*ḡ*_GABA_, *E*_GABA_, and excitability–affect PAD-induced spiking. We began with a minimalist conductance-based computer model to generate predictions that we then tested experimentally in acutely dissociated dorsal root ganglion (DRG) neurons using dynamic clamp. Intracellular recording/stimulation in most axons is prohibitively difficult but sustained depolarization of the soma or axon by optogenetic-based photostimulation evokes transient spiking, although photostimulation of peripheral axon terminals can evoke repetitive spiking in some DRG neurons [39]. It remains unclear how central axon terminals respond to sustained depolarization. We assume here that somatic and axonal spike initiation properties are qualitatively similar, but if axons were more excitable (i.e. more prone to repetitive spiking) than somata, they would operate farther to the right along the “excitability” axis described below. We applied virtual GABA conductances rather than assuming the soma and axon have equivalent GABA_A_R densities. As a final step, we confirmed our results in a multicompartment axon model.

### PAD-induced spiking in a model neuron

**Fig 1:**
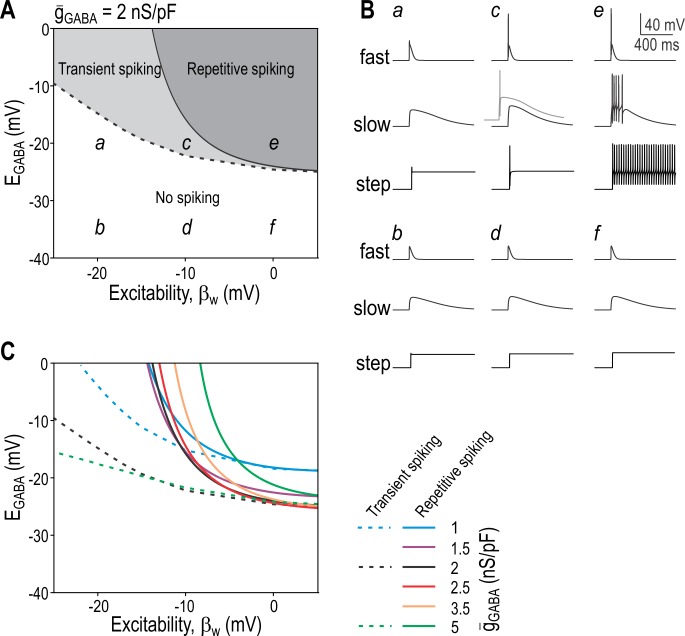
PAD-induced spiking in a neuron model. **(A)** 2-D bifurcation diagram showing the combinations of *E*_GABA_ and *β*_w_ that allow a GABA conductance step (*ḡ*_GABA_ = 2 nS/pF) to elicit repetitive or transient spiking (dark and light grey regions, respectively). Labels *a-f* indicate parameter combinations for which sample responses are shown in B. Normal conditions correspond to *E*_GABA_ = -35 mV and *β*_w_ = -20 mV (point *b*). **(B)** Sample responses to a *fast* synaptic waveform (*τ*_rise_ = 2 ms, *τ*_decay_ = 20 ms), a *slow* synaptic waveform (*τ*_rise_ = 20 ms, *τ*_decay_ = 200 ms) and a conductance *step* for parameter combinations labeled in A. Slow-onset conductance requires stronger *ḡ*_GABA_ (3.5 nS/pF, grey trace in *c*) to elicit transient spiking than a fast-onset conductance; all black traces are for *ḡ*_GABA_ = 2 nS/pF. **(C)** 2-D bifurcation analysis described in A was repeated for different *ḡ*_GABA_ values. Dashed and solid lines show borders for the transient and repetitive spiking regions, respectively. Increasing *ḡ*_GABA_ from 1 to 2 nS/pF (cyan → black) caused a downward shift in both borders but further increases (black → green) caused little change in the former and a rightward shift in the latter, indicating that increased *ḡ*_GABA_ confers increased spiking only to a certain point, beyond which further increase actually reduces spiking.

Starting with computer simulations, we co-varied *E*_GABA_ and intrinsic excitability (controlled by *β*_w_; see Methods) while keeping *ḡ*_GABA_ fixed at 2 nS/pF. The light grey and dark grey regions of the resulting 2-D bifurcation diagram (**Fig 1A**) show the *E*_GABA_ and *β*_w_ combinations that produce transient and repetitive spiking, respectively. Spiking pattern was determined by the response to GABA conductance “steps”. To more accurately simulate different forms of synaptic transmission, other conductance waveforms were tested: phasic inhibition via intrasynaptic GABA_A_R was modeled by a “fast” synaptic waveform (*τ*_rise_ = 2 ms; *τ*_decay_ = 20 ms; see Eq 6); tonic inhibition corresponds to the sustained component of the conductance step, but we also tested a “slow” synaptic waveform with intermediate kinetics to simulate spilled-over GABA asynchronously activating extrasynaptic GABA_A_R (*τ*_rise_ = 20 ms; *τ*_decay_ = 200 ms). **Fig 1B** shows responses to each stimulus waveform for parameter combinations labeled *a-f* on Fig 1A. Under control conditions used in the experiments described in this study (*E*_GABA_ = -35 mV and *β*_w_ = -20 mV; point *b*), GABA conductance caused depolarization but no spiking. PAD-induced repetitive spiking required a combined depolarizing shift in *E*_GABA_ and *β*_w_ (point *e*) whereas transient spiking required a smaller increase in *β*_w_ (point *c*) and could result solely from a large change in *E*_GABA_. By comparison, an isolated change in *β*_w_ could not enable PAD-induced spiking. As illustrated in panel *c* of Fig 1B, slow-onset GABA_A_R input required stronger input to elicit spiking because transient spiking involves a spike initiation mechanism that is sensitive to the rate of depolarization [40].

Next, we repeated the 2-D bifurcation analysis for different *ḡ*_GABA_ values to produce a family of curves (**Fig 1C**). The dashed curve demarcating the minimum requirements for transient spiking shifted downward as *ḡ*_GABA_ was increased. The solid curve demarcating the minimum requirements for repetitive spiking also shifted downward for an initial increase in *ḡ*_GABA_ but shifted rightward as *ḡ*_GABA_ was increased further, indicating that GABA_A_R activation is maximally excitatory at intermediate densities. Somatic recordings have demonstrated somatic *ḡ*_GABA_ between 0.2 and 0.5 nS/pF [35] and the absolute *ḡ*_GABA_ values reported by Chen et al. [13] correspond to approximately 0.1 nS/pF after conversion to densities based on estimated surface areas. Axonal *ḡ*_GABA_ may differ from somatic *ḡ*_GABA_ (given precedents for differential ion channel distribution [41]) but measuring *ḡ*_GABA_ in central axon terminals is prohibitively difficult. Our experimental approach does not rely on measuring axonal *ḡ*_GABA_ but, instead, was designed to determine the minimum *ḡ*_GABA_ required (for different *E*_GABA_ and intrinsic neuronal excitability) to enable PAD-induced spiking; comparing this value against measured *ḡ*_GABA_ (in the soma) reveals whether the density of native GABA receptors is sufficient to evoke spiking under different conditions. It remains unclear what *ḡ*_GABA_ would be necessary to evoke spiking in central axon terminals.

### PAD-induced transient spiking in DRG neurons

**Fig 2:**
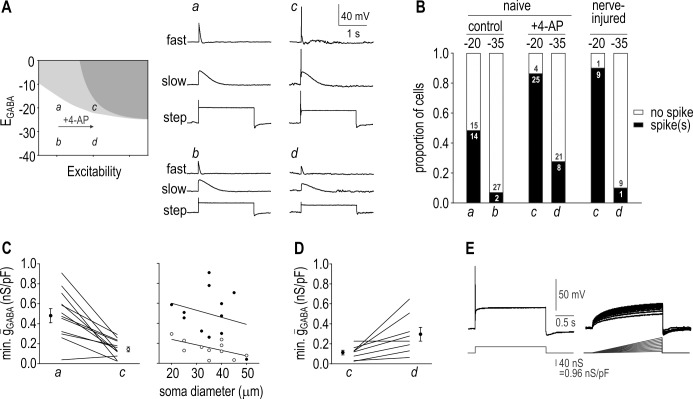
PAD-induced transient spiking in DRG neurons. **(A)** Sample responses to virtual GABA conductance applied via dynamic clamp. Labels *a-d* on cartoon indicate testing conditions and are referred to in all subsequent panels. Most neurons, like the typical one illustrated here, spiked only for *E*_GABA_ = -20 mV and after being made hyperexcitable by exposure to 2.5 mM 4-AP (point *c*). **(B)** Summary of the proportion of neurons responding with or without spikes to virtual PAD. Numbers inside each bar indicate the number of cells. A total of 29 neurons from naïve rats were tested before and after 4-AP and for each *E*_GABA_. A total of 10 neurons from nerve-injured rats were tested for each *E*_GABA_. The proportion of spiking/non-spiking cells was compared between conditions using Fisher’s exact tests (see Results). **(C)** Left panel summarizes the minimum *ḡ*_GABA_ required to elicit spiking in cells that spiked both before and after 4-AP for *E*_GABA_ = -20 mV. Minimum *ḡ*_GABA_ was significantly reduced from 0.76 ± 0.19 to 0.20 ± 0.05 nS/pF (mean ± SEM) by 4-AP (*p* = 0.005, two-way repeated measures ANOVA and Tukey test). These values are lower than observed in simulations in Fig 1; therefore, we adjusted the neuron model to reproduce this higher sensitivity to *ḡ*_GABA_. As illustrated in S1 Fig, this revised model shows the same relationship between *E*_GABA_ and excitability (*β*_w_) as seen in Fig 1. Right panel shows minimum *ḡ*_GABA_ plotted against soma diameter. Soma diameter, which correlates with fiber type, did not significantly affect minimum *ḡ*_GABA_ or the effect of 4-AP (*p* = 0.61 and 0.29, respectively; two-way repeated measures ANOVA). **(D)** Summary of the minimum *ḡ*_GABA_ required to elicit spiking in cells that spiked for each *E*_GABA_ value after 4-AP. The depolarizing shift in *E*_GABA_ from -35 mV to -20 mV caused a significant reduction (*p*<0.022, paired *t*-test) from 0.30 ± 0.07 nS/pF to 0.11 ± 0.02 nS/pF. **(E)** Sample responses from a typical neuron tested with *g*_GABA_ steps and ramps. The minimum *ḡ*_GABA_ required to elicit transient spiking when applied as a step was 40 nS (or 0.96 nS/pF after normalization by membrane capacitance) but a ramp with 2.5x greater peak amplitude failed to elicit spiking. *E*_GABA_ = -20 mV.

To test the simulation predictions described above, we conducted experiments in acutely dissociated DRG somata using an approach distinct from previous studies. Rather than activating native GABA_A_Rs by puffing GABA (which would produce a current whose conductance, reversal potential and kinetics are not easily measured or independently manipulated), we used dynamic clamp to apply a virtual conductance whose parameters are precisely and independently controllable. In this way, we quantified the minimum *virtual ḡ*_GABA_ required to elicit spiking under different conditions. Importantly, because virtual *ḡ*_GABA_ can be higher than native *ḡ*_GABA_, the density of native GABA_A_R does not limit our studies; indeed, failure of GABA puffs to evoke spikes in previous studies [13,35,42] suggests that somatic *ḡ*_GABA_ is normally too low to produce spikes, but *ḡ*_GABA_ may be higher in central axon terminals. In dynamic clamp, the voltage recorded from a neuron is passed to a computer, which, in real time, uses voltage to calculate current that is injected back into the patched neuron, thereby introducing a virtual conductance [43]. This approach allows manipulations to be applied like in computer simulations but to real neurons, such that we can avoid modeling the neuron (and making any assumptions about intrinsic excitability) and test directly how *virtual* GABA_A_R input affects *native* voltage-gated channels controlling spike initiation. Notably, photostimulation-based testing of axonal excitability has revealed transient spiking comparable to that observed in somata [39] but the excitability of central axon terminals remains uncertain. If central axon terminal and somatic excitability are similar, then the requirements for PAD-induced spiking ascertained for the soma can be extrapolated to those terminals; on the other hand, if those terminals are more excitable, they would operate farther to the right on the excitability axis depicted in the inset of **Fig 2A**.

To begin, we tested virtual GABA conductances in neurons from naïve animals before and after reproducing the hyperexcitability associated with nerve injury by blocking K_v_1-type potassium channels with 4-AP [44,45]; this corresponds in the model to setting *β*_w_ to less negative values. Testing with different *E*_GABA_ and stimulus waveforms, we systematically increased *ḡ*_GABA_ to try to elicit spiking. As illustrated for a typical cell in **Fig 2A,** PAD was most likely to produce spiking after application of 4-AP *and* a depolarizing shift in *E*_GABA_ to -20 mV. **Fig 2B** summarizes the proportion of cells in which PAD-induced spiking was observed under each test condition. For cells from naïve animals tested with *E*_GABA_ = -35 mV, 4-AP increased the proportion exhibiting PAD-induced spiking but not significantly (*p* = 0.079; Fisher’s exact test) whereas the 4-AP effect was highly significant for *E*_GABA_ = -20 mV (*p* = 0.004). Shifting *E*_GABA_ from -35 mV to -20 mV significantly increased the proportion of cells exhibiting PAD-induced spiking both before and after 4-AP (*p* < 10^−3^ and 10^−4^, respectively), consistent with the NKCC1 hypothesis of DRR generation [29,30]. But as predicted by our simulations, the proportion of cells with PAD-induced spiking was most significantly increased by the combination of 4-AP and a depolarizing shift in *E*_GABA_ (*p* < 10^−9^). Within this data set, two cells were subsequently identified as outliers based on analysis of the minimum *ḡ*_GABA_ needed for PAD-induced spiking (see below); removing those outliers did not substantively alter the statistical results reported above.

Based on cells that exhibited PAD-induced spiking before and after 4-AP for *E*_GABA_ = -20 mV, the minimum *ḡ*_GABA_ needed to elicit spiking was significantly reduced from 0.49 ± 0.07 nS/pF (mean±SEM) to 0.16 ± 0.03 nS/pF by 4-AP (*p* = 0.005, Tukey test following ANOVA described below) (**Fig 2C left**). Plotting the same data against soma diameter revealed a trend towards higher minimum *ḡ*_GABA_ for smaller cells, but soma diameter did not have a significant effect (*p* = 0.61) and nor did it interact significantly with the 4-AP effect (*p* = 0.29; two-way repeated measures ANOVA) (**Fig 2C right**). Notably, we report all conductances as densities to correct for the direct effect of membrane surface area on our measurements; however, soma diameter is known to correlate with fiber type [46], and so the insignificant effect of cell size (after normalization by surface area) argues that minimum *ḡ*_GABA_ does not differ significantly between myelinated (A) and unmyelinated (C) neurons. Of the cells that exhibited PAD-induced spiking for both *E*_GABA_ values after 4-AP, the minimum *ḡ*_GABA_ needed to elicit spiking was significantly reduced from 0.30 ± 0.07 nS/pF to 0.11 ± 0.02 nS/pF by shifting *E*_GABA_ from -35 mV to -20 mV (*p* = 0.022, paired *t*-test) (**Fig 2D**). Of the 10 cells tested with both fast and slow *g*_GABA_ waveforms at *E*_GABA_ = -20 mV after 4-AP, 7 responded to both stimuli with transient spiking and 2 responded with repetitive spiking. Among transient spiking cells, the slow waveform required higher *ḡ*_GABA_ than the fast waveform to elicit transient spiking (0.46 ± 0.09 nS/pF vs 0.27 ± 0.09 nS/pF) which, although not a statistically significant difference (*p* = 0.25; paired *t*-test), is consistent with a spike initiation mechanism sensitive to the rate of depolarization. By comparison, the two repetitive spiking cells required exactly the same minimum *ḡ*_GABA_ for the fast and slow waveforms, consistent with a spike initiation mechanism sensitive only to the amplitude of depolarization [40]. Comparing the responses to *g*_GABA_ steps and ramps illustrates that the latter are far less effective in eliciting transient spiking (**Fig 2E**). All of these experimental data are consistent with simulation results in Fig 1 and S1 Fig.

Like 4-AP, nerve injury increased the proportion of cells exhibiting PAD-induced spiking (bars on right side of **Fig 2B**). Compared against naïve cells without 4-AP, nerve injury caused no change in the proportion of cells exhibiting PAD-induced spiking for *E*_GABA_ = -35 mV (*p* = 1) whereas it did significantly increase that proportion for *E*_GABA_ = -20 mV (*p* = 0.028; Fisher’s exact tests). Nerve injury and treatment of naïve cells with 4-AP resulted in a similar proportion of cells with PAD-induced spiking when tested with *E*_GABA_ = -35 mV and -20 mV (*p* = 1 and 0.40, respectively). Among nerve-injured cells, shifting *E*_GABA_ from -35 mV to -20 mV significantly increased the proportion with PAD-induced spiking (*p* = 0.001). Consistent with the combined effects of 4-AP and altered *E*_GABA_, the proportion of cells with PAD-induced spiking was most significantly increased by the combination of nerve injury and a depolarizing shift in *E*_GABA_ (*p* < 10^−5^).

### PAD-induced repetitive spiking in DRG neurons

**Fig 3:**
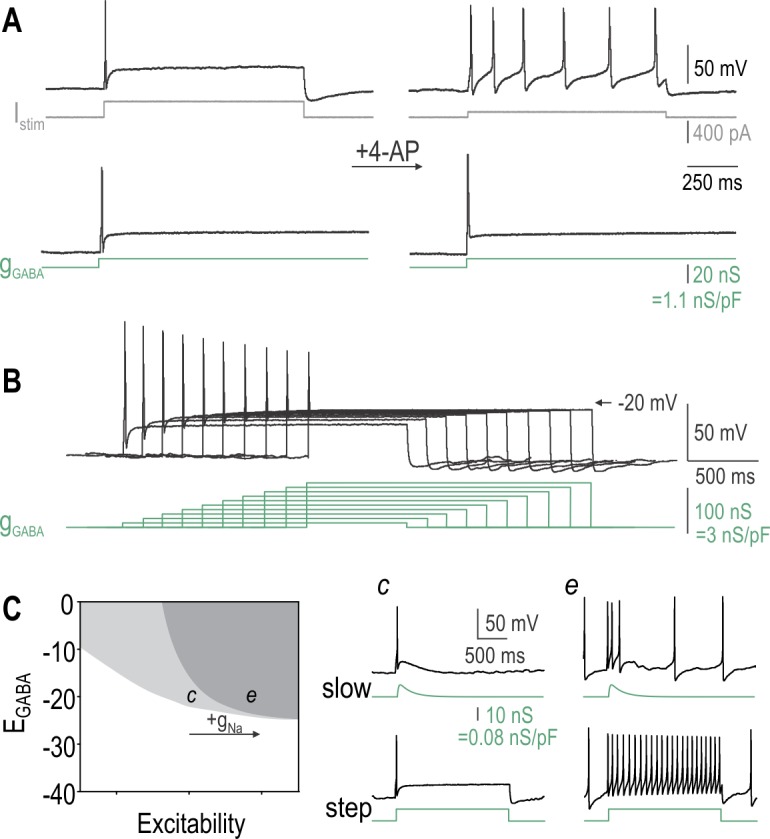
PAD-induced repetitive spiking in DRG neurons. **(A)** Sample traces from a typical neuron showing that 4-AP had the intended effect of enabling repetitive spiking in response to current injection (*I*_stim_, top traces), yet virtual GABA conductance continued to elicit only transient spiking (bottom traces). *E*_GABA_ = -20 mV. Scale bar for *g*_GABA_ in all panels show nS before and after normalization by membrane capacitance of the recorded cell. **(B)** Responses from another neuron showing that increasing *ḡ*_GABA_ across a very broad range (an order of magnitude greater than required for transient spiking) failed to eventually induce repetitive spiking. Instead, spike amplitude was attenuated and membrane potential was effectively clamped near *E*_GABA_ after the initial spike. **(C)** To further increase excitability, dynamic clamp was used to insert a virtual voltage-dependent sodium conductance (*ḡ*_Na_ = 0.2 nS/pF) after applying 4-AP. The effectiveness of this manipulation is clear from the development of spontaneous spiking (right panels). Under these conditions, a slow *g*_GABA_ waveform or *g*_GABA_ step induced repetitive spiking. The result was observed in 2 of 2 neurons tested.

Testing with current injection (*I*_stim_) confirmed that 4-AP had the intended effect of increasing excitability yet, despite responding to *I*_stim_ steps with repetitive spiking, most neurons responded to *g*_GABA_ steps with transient spiking, as illustrated in **Fig 3A**. Specifically, PAD-induced repetitive spiking was not observed in any nerve-injured neurons and was seen in only two neurons after 4-AP application. All neurons were tested with a broad range of *ḡ*_GABA_ to confirm that repetitive spiking could not eventually be achieved by applying a stronger conductance. Increasing *ḡ*_GABA_ above the minimum required to elicit transient spiking consistently caused attenuation of the spike height and clamped the subsequent voltage near *E*_GABA_ (**Fig 3B**). Based on our simulation results (see Fig 1A), we reasoned that the lack of repetitive spiking was due to 4-AP or nerve injury not causing a sufficient increase in excitability. To test this hypothesis, we further increased excitability by using dynamic clamp to introduce a virtual sodium conductance like that upregulated after nerve injury [45]. As predicted, PAD-induced repetitive spiking was made possible by this additional manipulation (**Fig 3C**). Although we managed to reproduce PAD-induced repetitive spiking, the extent of the required manipulations suggests that naturally occurring pathological changes cause few neurons to become sufficiently hyperexcitable that PAD will induce repetitive spiking. That said, if the central terminals of axons are more excitable (i.e. more prone to repetitive spiking) than the soma, PAD would be more likely to elicit repetitive spiking than suggested by our data.

### PAD-mediated inhibition in a model neuron

The above results demonstrate that depolarizing GABA current can induce transient spiking under conditions associated with nerve injury. This does not, however, exclude PAD from retaining its inhibitory effects, especially given that inhibition stems from sodium channel inactivation and shunting. In fact, although PAD may induce a single spike at its onset, shunting effects persist as long as GABA_A_R are activated. This raises the important question of whether more spikes (arising in the periphery or ectopically in the soma or a neuroma) are blocked by PAD than are induced by PAD in the central axon terminals.

**Fig 4:**
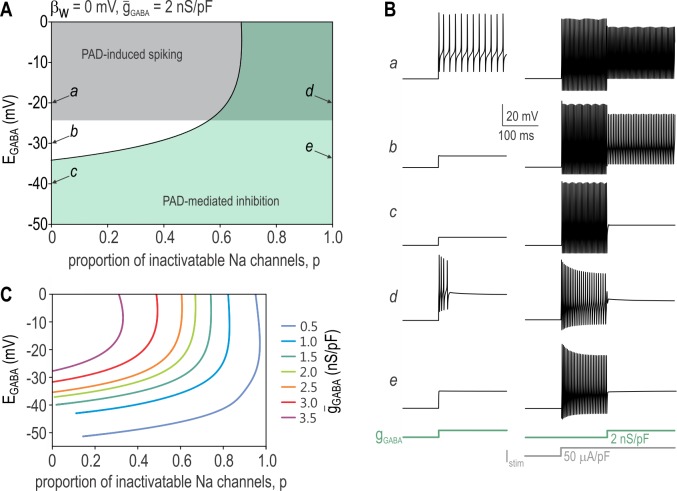
PAD-mediated inhibition in a neuron model. **(A)** 2-D bifurcation diagram showing the combinations of *E*_GABA_ and *p* associated with PAD-induced spiking (grey region) and PAD-mediated inhibition of other spiking (green region), where *p* represents the proportion of sodium channels susceptible to inactivation. Simulations here are based on a neuron model with sodium channel inactivation (Eq 7) with *β*_w_ = 0 mV and *ḡ*_GABA_ = 2 nS/pF. Note that the green and grey regions overlap, indicating that PAD can initiate its own spikes yet still inhibit spikes initiated by other means (e.g. by stimulating current *I*_stim_). Labels *a-e* indicate parameter combinations for which sample responses are shown in B. **(B)** Responses to *g*_GABA_ steps occurring alone or during *I*_stim_ steps are shown down the left and right columns, respectively. **(C)** Boundary between inhibitory and non-inhibitory region (as in A) re-plotted for different *ḡ*_GABA_. Higher *ḡ*_GABA_ enables GABA to be inhibitory despite less inactivating sodium current (i.e. smaller *p*) and more depolarized *E*_GABA_.

Our initial model did not include sodium channel inactivation for the sake of simplicity; therefore, our next step was to modify the model so that a certain proportion of sodium channels, controlled by parameter *p*, experience inactivation (Eqn. 7). Using this new model, we set *β*_w_ to 0 mV to facilitate repetitive spiking and conducted 2-D bifurcation analysis to determine the *p* and *E*_GABA_ combinations associated with different effects of PAD (**Fig 4A**). The grey region shows parameter combinations for which a *g*_GABA_ step (2 nS/pF) applied alone elicits spiking (sample traces *a* and *d* in **Fig 4B**). The green region shows parameter combinations for which the same *g*_GABA_ step inhibits spiking induced by *I*_stim_ steps (sample traces *c*-*e* in **Fig 4B**). Importantly, the green and grey regions overlap, thus demonstrating that PAD can induce spikes yet nonetheless block spikes originating by other means. **Fig 4C** shows the 2-D bifurcation analysis repeated for different *ḡ*_GABA_ values. The region of PAD-induced spiking remained unchanged (not illustrated) but the region of PAD-mediated inhibition expanded as *ḡ*_GABA_ was increased, suggesting that stronger GABA_A_R activation manages to terminate spiking despite a smaller proportion of inactivatable sodium channels.

### PAD-mediated inhibition in DRG neurons

**Fig 5:**
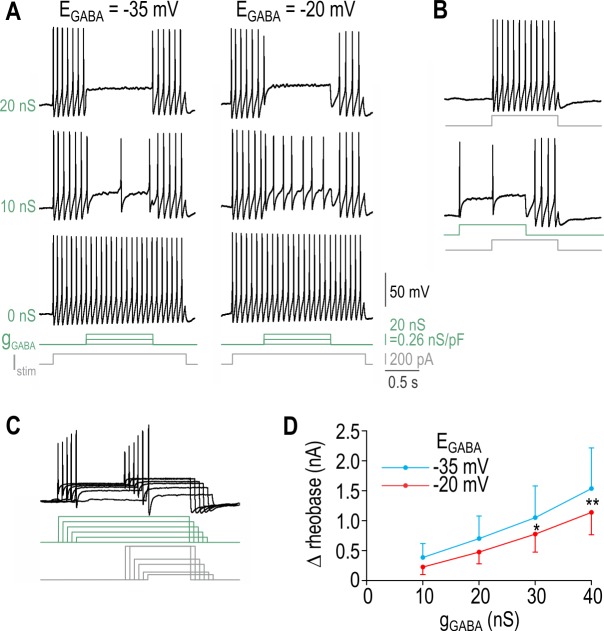
PAD-mediated inhibition in DRG neurons. **(A)** Sample responses from a typical neuron made hyperexcitable by 4-AP and virtual sodium conductance (*ḡ*_Na_ = 0.3 nS/pF). The repetitive spiking elicited by the *I*_stim_ step was reduced by application of a small *g*_GABA_ step (middle row) and was altogether stopped by larger *g*_GABA_ steps (top row). For equivalent *ḡ*_GABA_, stronger inhibition was evident with *E*_GABA_ = -35 mV than with *E*_GABA_ = -20 mV (compare left and right columns). **(B)** Sequence of *I*_stim_ and *g*_GABA_ steps was changed to verify that the latter could elicit transient spiking yet still inhibit the repetitive spiking driven by *I*_stim_. Note that repetitive spiking starts after the *g*_GABA_ step ends and lasts until the *I*_stim_ step ends. **(C)** PAD-mediated inhibition of transient spiking was assessed using the same protocol as in B but we varied the amplitude of the *I*_stim_ step to determine rheobase (i.e. the minimum *I*_stim_ required to evoke spiking). Only responses to rheobasic stimulation are shown. Note that rheobase increases with increases in *ḡ*_GABA_, whereas spike height decreases. **(D)** Change in rheobase (mean ± SEM) is plotted against *ḡ*_GABA_ for *E*_GABA_ = -35 mV (blue, *n* = 3 cells) and -20 mV (red, *n* = 4 cells). Rheobase was significantly increased by *ḡ*_GABA_ (*p* = 0.013, one-way repeated measures ANOVA; *p* = 0.013 (*), *p* = 0.002 (**), Holm-Sidak post-hoc tests vs no *g*_GABA_) but *E*_GABA_ did not have a significant effect (*p* = 0.52).

To measure PAD-mediated inhibition in real DRG neurons, we combined *g*_GABA_ and *I*_stim_ steps as done for simulations in Fig 4B. **Fig 5A** shows a typical neuron in which *I*_stim_ elicited repetitive spiking. Interposing a *g*_GABA_ step during the *I*_stim_ step caused reduction or complete cessation of repetitive spiking depending on *ḡ*_GABA_ and *E*_GABA_. Note that spikes occurring during the *g*_GABA_ step were shorter than those occurring outside the *g*_GABA_ step, consistent with the shunting effect of the virtual GABA conductance. Applying the *g*_GABA_ step before the onset of *I*_stim_ confirmed that the former could elicit transient spiking yet still inhibit the repetitive spiking otherwise driven by *I*_stim_ (**Fig 5B**). Using the same stimulus sequence, we measured rheobase (i.e. the minimum *I*_stim_ required to elicit spiking) for each level of *ḡ*_GABA_ (**Fig 5C**). Rheobase was significantly increased by increments in *ḡ*_GABA_ (*p* = 0.013, two-way repeated measures ANOVA) but was not significantly affected by *E*_GABA_ (*p* = 0.52) (**Fig 5D**). These data confirm that PAD elicited in the cell body of DRG neurons mediates shunting inhibition even under conditions in which it can induce spiking.

### Possible involvement of calcium-activated chloride channels in PAD

**Fig 6:**
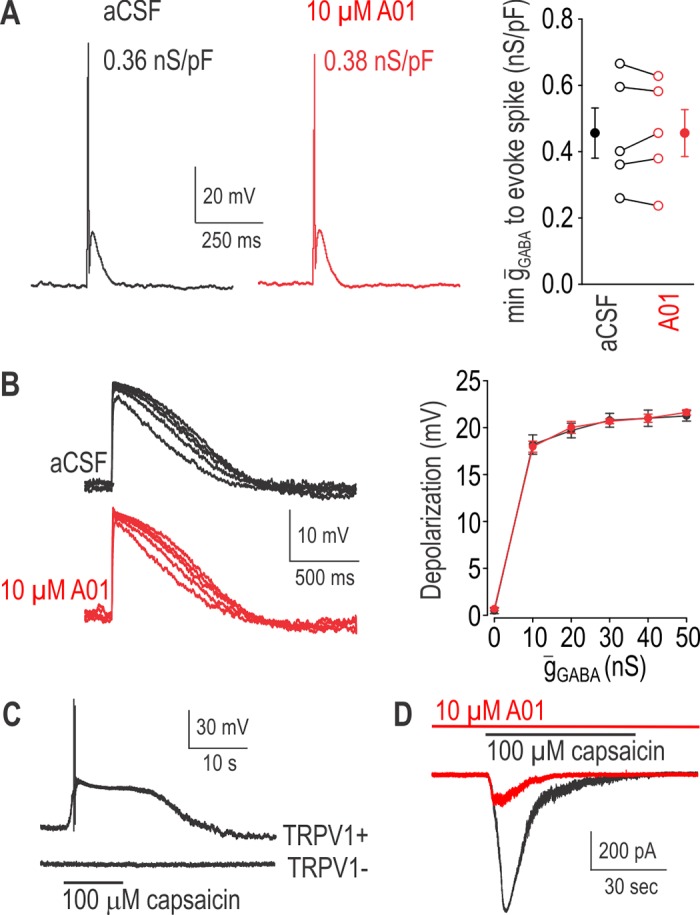
ANO-1 channels do not contribute to PAD. For all panels, responses in the presence of the ANO-1 antagonist T16Ainh-A01 (A01) are shown in red for comparison against responses in normal aCSF shown in black. **(A)** Traces show responses in a typical neuron to the minimum virtual *ḡ*_GABA_ required to evoke spiking based on a fast synaptic waveform and *E*_GABA_ = -20 mV before and after ANO-1 blockade. Summary data show that the minimum *ḡ*_GABA_ to evoke spiking was not significantly changed by A01 (*p* = 1.0; paired *t*-test) based on all TRPV1+ neurons (*n* = 5) that spiked in response to virtual GABA. **(B)** Traces show responses in a typical neuron to different *ḡ*_GABA_ based on slow synaptic waveform and *E*_GABA_ = -35 mV. Summary data show mean (± SEM) depolarization at different *ḡ*_GABA_ for all (*n* = 7) TRPV1+ neurons tested. Blockade of ANO-1 did not significantly affect depolarization (*p* = 0.77; two-way repeated measure ANOVA). **(C)** At the end of each experiment, the recorded cell was stimulated with capsaicin. Traces show typical data from a responsive (TRPV1+) and unresponsive (TRPV1-) neuron. Because ANO-1 is expressed predominantly in TRPV1+ neurons, only data from capsaicin-responsive neurons were included for analysis in panels A and B. **(D)** To confirm the efficacy A01, we verified that it reduced the response to capsaicin, consistent with Takayama et al. [49].

Activation of the calcium-activated chloride channel ANO-1 in primary afferent neurons can evoke or exacerbate pain, especially under inflammatory or neuropathic conditions [47–50]. Notably, intracellular chloride tends to be elevated under those conditions (see Introduction), which may explain why ANO-1 activation is excitatory rather than inhibitory. Consistent with this, ANO-1 modulation of spiking evoked by current injection is sensitive to intracellular chloride level [51] but demonstration that ANO-1 itself evokes spiking was based on a chloride reversal potential of -18 mV [49]. Given its activation requirements [52], we predicted that ANO-1 channels would not be activated by the GABAergic input underlying PAD; recall that GABA_A_R activation is necessary for PAD [22]. Nonetheless, to rule out a contribution by ANO-1, we repeated virtual PAD experiments (like in Fig 2) before and after blockade of ANO-1 channels by bath-applied 10 μM T16Ainh-A01 (A01) (**Fig 6**). Based on the pipette solution, the chloride reversal potential for ANO-1 was -20 mV but *E*_GABA_ for virtual *g*_GABA_ was set to -35 or -20 mV in dynamic clamp. As predicted, ANO-1 blockade had no significant effect on the minimum *ḡ*_GABA_ needed to evoke spiking for *E*_GABA_ = -20 mV (*p* = 1.0, paired *t*-test; **Fig 6A**) and nor did it significantly affect the depolarization evoked by different *ḡ*_GABA_ for *E*_GABA_ = -35 mV (*p* = 0.77, two-way repeated measures ANOVA; **Fig 6B**) or have any effect on rheobase, input resistance, or resting membrane potential (*p* > 0.05, paired *t*-tests). The data above are based exclusively on capsaicin-responsive cells (see **Fig 6C**) since ANO-1 channels are expressed primarily in cells that express TRPV1 [47]. Notably, the response to capsaicin was reduced by ANO-1 blockade (**Fig 6D**), consistent with Takayama et al. [49] and thus verifying the efficacy of our A01. Based on these results, we conclude that ANO-1 channels are not activated and, therefore, do not contribute to PAD under our experimental conditions.

### PAD-mediated inhibition of spike propagation in a multicompartment model neuron

**Fig 7:**
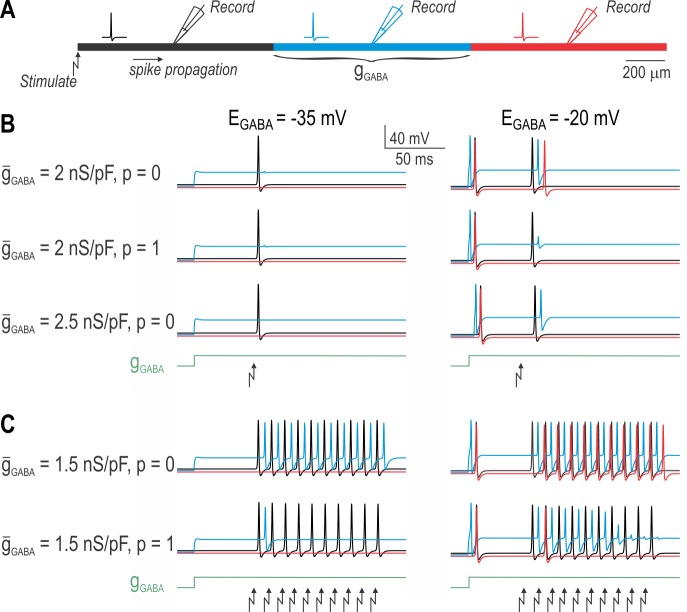
PAD-mediated inhibition of spike propagation in a multi-compartment axon model. **(A)** Cartoon depicts our three-compartment axon model. One or more spikes were initiated by current injection applied to the left end of the axon. Voltage was measured at the midpoint of each compartment; color of traces in B and C correspond to compartment colors shown in A. GABA conductance was distributed uniformly throughout the middle (blue) compartment. **(B)** For *E*_GABA_ = -35 mV (left column), *g*_GABA_ blocked the propagation of the evoked spike under all three combinations of *ḡ*_GABA_ and *p* that were tested, where *p* represents the proportion of sodium channels susceptible to inactivation. The *g*_GABA_ step did not elicit its own spiking in any condition. On the other hand, for *E*_GABA_ = -20 mV (right column), PAD-induced transient spiking was observed for all three conditions yet propagation of the stimulus-evoked spike was blocked in two of the three conditions. Comparing the top and middle panels shows that modest *ḡ*_GABA_ relies on sodium channel inactivation to block spike propagation, whereas stronger *ḡ*_GABA_ could block propagation without any contribution from sodium channel inactivation. **(C)** During a spike train, sodium channel inactivation accumulates between spikes such that spikes early in the train can propagate whereas later spikes do not. Comparing with combinations of *ḡ*_GABA_ and *p* required to block propagation of a single spike (see B), these results show that partial blockade during a spike train can be mediated by even comparatively weak PAD.

All simulations described thus far were conducted in a single compartment model. This adequately simulates spike initiation occurring in proximity to the recording electrode, as occurs when recording from an isolated DRG soma. Although spontaneous or PAD-induced spiking may arise at the site of PAD, an important inhibitory effect of PAD in the intact fiber is to block the orthodromic propagation of spikes originating in the periphery. To test for conduction block, we converted our single-compartment model into a 3-compartment model (**Fig 7A**). Although still very simple compared with past models used to study this topic [e.g. 19,25,53], this model suffices to qualitatively illustrate key points relevant for the present study. Each compartment was further subdivided into equipotential segments. Based on its small diameter and the absence of nodes, this model simulates continuous propagation in an unmyelinated fiber. By applying GABA conductance to the middle compartment, we tested if that conductance can induce spikes (originating within that compartment) and/or block the propagation of other spikes (evoked at the far end of adjacent compartment).

For an *E*_GABA_ value of -35 mV, *g*_GABA_ never evoked spiking (consistent with the single-compartment model) but it did block spike propagation (**Fig 7B**, left column). Interestingly, blocked propagation could occur even in the absence of sodium channel inactivation, therein supporting claims that shunting mediated by *g*_GABA_ mediates an inhibitory effect. When *E*_GABA_ was shifted to -20 mV, *g*_GABA_ evoked a single spike that propagated in both directions away from the center compartment (**Fig 7B**, right column). Yet despite this excitatory effect, propagation of other spikes was blocked in two of the three conditions illustrated. Sample traces were chosen to illustrate that large *g*_GABA_ could block propagation in the absence of sodium channel inactivation but a smaller *g*_GABA_ could achieve the same effect when combined with sodium channel inactivation. **Fig 7C** demonstrates that sodium channel inactivation can accumulate over time, thus eventually blocking spikes traveling as part of a train. These results confirm that PAD does not abruptly lose its inhibitory effects once able to induce its own spikes.

## Discussion

Using computer simulations and an experimental approach distinct from previous studies, we have identified which pathological changes are necessary and sufficient to enable PAD-induced spiking. We determined that a depolarizing shift in *E*_GABA_ is necessary yet insufficient to enable PAD-induced spiking in most DRG neurons. An increase in intrinsic excitability (i.e. altered spike initiation properties) is also necessary, especially to enable PAD-induced repetitive spiking. Neurons may experience both changes after nerve injury or inflammation, meaning PAD-induced spiking could occur in certain pathological conditions [22,26,29]; however, other factors such as the requirement for fast depolarization suggest that PAD-induced spiking is probably rare (see below), but this depends on the excitability of central axon terminals, which still remains uncertain. Intriguingly, our data also suggest that PAD continues to mediate presynaptic inhibition under conditions in which it can induce transient spiking. Although seemingly paradoxical, the co-existence of excitatory and inhibitory effects has been observed previously in studies of presynaptic inhibition in crayfish [54] and is consistent with the biophysical mechanisms responsible for each effect. This is unlike what happens postsynaptically in spinal neurons, where paradoxical excitation develops only after inhibition fails [10,55]. Our data argue that increased PAD has a net inhibitory effect, meaning paradoxical excitation via enhanced PAD poses less risk to somatosensory processing than disinhibition caused by reduced PAD.

The GABA conductance density required for PAD-induced spiking under normal conditions is evidently quite high, so much so that we were able to elicit spiking in only 2 of 29 neurons despite testing with virtual *ḡ*_GABA_ several times greater than the typical density measured in somata [13,35]. This is consistent with previous failures to elicit spikes by puffing GABA on the soma [13,35,42]. Puffed GABA also failed to elicit calcium signals when applied to the central terminals of GCaMP-expressing primary afferents [13], and Verdier et al. [56] observed GABA-induced spiking in only 4 of 77 neurons tested in the trigeminal nucleus. The value of *ḡ*_GABA_ in central axon terminals remains an open question but evidence points to reduced expression of presynaptic GABA_A_Rs following nerve injury [13,57,58], which suggests that presynaptic inhibition is weakened by reduction of *ḡ*_GABA_ rather than *ḡ*_GABA_ becoming strong enough that PAD induces spiking. That said, the minimum *ḡ*_GABA_ needed for PAD-induced spiking is reduced by increased neuronal excitability (**Fig 2C**) and by a depolarizing shift in *E*_GABA_ (**Fig 2D**). Unlike an increase in *ḡ*_GABA_, which increases inhibitory effects due to shunting, increased neuronal excitability and depolarized *E*_GABA_ can encourage PAD-induced spiking without enhancing PAD-mediated shunting. Studying transient spiking cells in the chick cochlear nucleus, Monsivais and Rubel [59] found that depolarizing GABA_A_R input could elicit spiking after blockade of the low-threshold potassium current known to be responsible for transient spiking [60]. The same GABA_A_R input normally inhibited stimulus-evoked spiking by activating the low-threshold potassium current and thereby elevating spike threshold [59]. Those data are entirely consistent with results presented here. Notably, PAD-induced spiking would be more likely in central axon terminals if those terminals are more excitable that we have assumed based on extrapolation from somatic data.

Intracellular chloride *could* be depleted during PAD if chloride uptake via NKCC1 became saturated (at least transiently) and thus failed to keep pace with chloride efflux via activated GABA_A_ channels. The potential for altered chloride concentration is exacerbated by the small caliber of central axon terminals, especially C fibers, since intracellular volume is small compared to surface area [61]. Chloride depletion, if it occurred, would cause an activity-dependent hyperpolarizing shift in *E*_GABA_, the implication being that *E*_GABA_ may be near -20 mV only at the onset of GABA_A_R activation. Given that PAD-induced spiking depends on a depolarized *E*_GABA_ value, a hyperpolarizing shift would discourage PAD-induced repetitive spiking. That said, the transient spiking observed in our dynamic clamp experiments was not due to chloride depletion since the virtual GABA current is mediated by current injection through the patch pipette rather than by chloride efflux across the cell membrane. In effect, PAD-induced repetitive spiking may be more difficult to evoke under natural conditions, and transient spiking may rely even more heavily on abrupt depolarization than our experiments suggest.

Following on the above points, both simulations and experiments demonstrated that smaller pathological changes in *E*_GABA_ and/or excitability are required to enable PAD-induced transient spiking than are required for PAD-induced repetitive spiking. This has important implications. Even if sustained, PAD is likely to produce only one spike at its onset (if it produces any spikes at all) and will likely not produce any spikes unless its onset is abrupt. This is because transient spiking involves a spike initiation mechanism that is sensitive to the rate of depolarization [40]. Sensitivity to *g*_GABA_ onset kinetics would be inconsequential if presynaptic inhibition was phasic, which is to say that the GABA_A_Rs are clustered within the synaptic cleft and therefore receive an abrupt pulse of GABA upon its vesicular release [62], but evidence points toward a more tonic mode of action (unlike the phasic inhibition studied in the crayfish neuromuscular junction [63]) as outlined below. Recording from mammalian primary afferent terminals to measure the activation kinetics (and density) of the GABA_A_R current is prohibitively difficult, but immunocytochemical evidence argues that C fiber terminals are devoid of gephyrin [64]. Since gephyrin is usually necessary for GABA_A_R clustering [65], its absence suggests that GABA_A_Rs are distributed more diffusely. Electrophysiological evidence for high-affinity GABA_A_Rs in primary afferent neurons [37] supports this view since such receptors have a δ subunit [66] in place of the γ subunit that is necessary for clustering [62,67]. If primary afferent GABA_A_Rs are indeed distributed extrasynaptically, and are thus activated asynchronously as GABA diffuses beyond the synaptic cleft, then *g*_GABA_ will have slow onset kinetics and is unlikely to elicit transient spiking. Only the most hyperexcitable fibers (i.e. those capable of PAD-induced repetitive spiking) are likely to exhibit any PAD-induced spiking. And whereas PAD-induced transient spiking relies on abrupt GABA_A_R activation, PAD-mediated inhibition does not; instead, PAD-mediated inhibitory effects will last throughout the duration of the PAD. In other words, slow activation of extrasynaptic GABA_A_Rs–arguably the most likely scenario at least for C fiber terminals (see above)–will not cause PAD-induced spiking but will cause PAD-mediated inhibition.

Notably, dorsal root reflexes (DRRs) have typically been studied using electrical stimulation of a nerve or dorsal root to synchronously activate a large number of afferent fibers [e.g. 68]. Notwithstanding differential conduction latencies, such input will evoke a large burst of GABA release, causing GABA_A_R activation that is ideally suited for PAD-induced transient spiking. It is not obvious that those same fibers would exhibit PAD-induced spiking under more natural conditions (i.e. less synchronous inputs). However, Dubuc et al. [69] observed antidromic spiking in 19% of cat dorsal root fibers during fictive locomotion. It has long been recognized that dorsal root reflexes are more common in certain afferents (e.g. stretch receptors) with direct evidence for DRRs being weakest in C fibres [22]. However, Lin et al. [36] reported spontaneous and von Frey-evoked antidromic spiking in all fiber types and, moreover, found that intradermal capsaicin selectively increased antidromic spiking in C and Aδ fibers. Based on more recent observations, including data presented here, one may suspect that chloride regulation, GABA receptor clustering and/or intrinsic excitability differ between afferent types. Somatic recordings suggest that important differences do indeed exist [70] but definitively resolving this requires comparison of axon terminals (rather than somata) and is therefore technically difficult. Notably, Dubuc et al. [69] observed repetitive antidromic spiking, as have others [e.g. 71], which argues that the excitability of certain afferent terminals is quite high. The role of axonal excitability warrants closer attention in future studies. Observation that cooling increases DRRs [72] likely holds important clues. Please see [5] for a recent review of other factors.

As already explained, PAD-induced spiking does not equate with failure of presynaptic inhibition. The resilience of presynaptic inhibition is best appreciated by comparing how pre- and postsynaptic inhibition fail. As KCC2 is downregulated postsynaptically, *E*_GABA_ undergoes a depolarizing shift that directly compromises the inhibitory effect of GABAergic input [61]. The same shift in *E*_GABA_ that reduces postsynaptic inhibition is what eventually results in paradoxical excitation. This shift from inhibition to paradoxical excitation is evidently not what happens presynaptically. In primary afferent terminals, the changes required for paradoxical excitation–a shift in *E*_GABA_ and increased excitability–do not undermine the inhibitory effect; in fact, the relatively high *ḡ*_GABA_ required for PAD-induced spiking also encourages PAD-mediated inhibition. This conclusion contradicts past assumptions on this matter. Furthermore, whereas the risk of paradoxical excitation increases postsynaptically during sustained GABAergic input (because of chloride accumulation), presynaptically, the balance shifts towards inhibitory effects over time as sodium channel inactivation accumulates and if intracellular chloride is depleted. The greatest risk to presynaptic inhibition is reduced PAD rather than enhanced PAD.

To conclude, we have demonstrated that combined changes in *E*_GABA_ and intrinsic excitability enable PAD-induced transient spiking. However, unless neurons become so hyperexcitable that PAD can induce repetitive spiking, slow (asynchronous) activation of extrasynaptic GABA_A_Rs is unlikely to elicit any spiking. On the other hand, PAD will continue to mediate presynaptic inhibition. In practical terms, our results suggest that presynaptic inhibition is a viable therapeutic target whose enhancement carries little risk of causing paradoxical excitation.

## Methods

### Ethics Statement

All experiments were approved by the University of Pittsburgh IACUC and by The Hospital for Sick Children Animal Care Committee.

### Computer simulations

Starting from a previously published model [45,73], our single compartment, conductance-based model is described as follows:

$$C\;\frac{dV}{dt}=I_{\text{stim}}-{\bar{g}}_{\text{Na}}m_{\infty }\left(V\right)\left(V-E_{Na}\right)-{\bar{g}}_{\text{K}}w\left(V-E_{\text{K}}\right)-g_{\text{leak}}\left(V-E_{\text{leak}}\right)-g_{\text{GABA}}\left(t\right)\left(V-E_{\text{GABA}}\right)$$ (1)

where activation variable *m* changes instantaneously with voltage *V* according to

$$m_{\infty }\left(V\right)=0.5\left[1+\tanh\left(\frac{V-\beta_{\text{m}}}{\gamma_{\text{m}}}\right)\right],$$ (2)

whereas *w* changes more slowly according to

$$\frac{dw}{dt}=\phi_{\text{w}}\frac{w_{\infty }\left(V\right)-w}{\tau_{w}\left(V\right)},$$ (3)

$$w_{\infty }\left(V\right)=0.5\left[1+\tanh\left(\frac{V-\beta_{\text{w}}}{\gamma_{\text{w}}}\right)\right],$$ (4)

$$\tau_{w}\left(V\right)=\frac{1}{\cosh\left(\frac{V-\beta_{\text{w}}}{2\gamma_{\text{w}}}\right)}.$$ (5)

Neuronal excitability was varied by changing parameter *β*_w_ [38]. Injury-induced hyperexcitability can be reproduced by shifting *β*_w_ from its normal value of around -20 mV to less negative values [73]. Setting *β*_w_ to less negative values reflects a multitude of potential injury-induced molecular changes including reduced K_V_1-type potassium current, which we model experimentally using 4-AP application, and increased sodium current, which we model experimentally using dynamic clamp (see below); the effect of such changes, occurring alone or together, is to alter spike initiation [45]. All other neuronal parameters were fixed as reported previously [38] at the following values: *C* = 2 μF/cm^2^; sodium conductance *ḡ*_Na_ = 20 mS/cm^2^, *E*_Na_ = 50 mV, *β*_m_ = -1.2 mV, *γ*_m_ = 18 mV; potassium conductance *ḡ*_K_ = 20 mS/cm^2^, *E*_K_ = -100 mV, *ϕ*_*w*_ = 0.15, *γ*_w_ = 10 mV; leak conductance *g*_leak_ = 2 mS/cm^2^, *E*_leak_ = -70 mV.

Stimulating current *I*_stim_ was not applied unless indicated. Maximal GABA conductance density *ḡ*_GABA_ and reversal potential *E*_GABA_ were varied. Units for *ḡ*_GABA_ were converted to nS/pF for comparison with experimental measurements. The normal *E*_GABA_ value in primary afferent is around -35 mV based on measurements using different techniques [12,13,35,42]. GABA conductance was activated as a step or as a synaptic waveform described by

gGABA(t)=g¯GABAx[−e−tτrise+e−tτdecay], (6)

which comprises an exponential rise to maximum (with time constant *τ*_rise_) followed by an exponential decay back to baseline (with *τ*_decay_). The peak is normalized to 1 by factor *x* before being scaled by *ḡ*_GABA_. Kinetics are reported in the Results section.

For simulations reported in Figs 4 and 7, sodium channel inactivation *h* was applied to a proportion of sodium channels defined by *p*, thus giving the following current balance equation

$$C\;\frac{dV}{dt}=I_{\text{stim}}-p\;{\bar{g}}_{\text{Na}}m_{\infty }\left(V\right)h\left(V-E_{Na}\right)-\left(1-p\right)\;{\bar{g}}_{\text{Na}}m_{\infty }\left(V\right)\left(V-E_{Na}\right)-{\bar{g}}_{\text{K}}w\left(V-E_{\text{K}}\right)-g_{\text{leak}}\left(V-E_{\text{leak}}\right)-g_{\text{GABA}}\left(t\right)\left(V-E_{\text{GABA}}\right).$$ (7)

Changes in *h* are described by the same equations used to describe *w* (Eq 3–5) where *β*_h_ = -28 mV, *γ*_h_ = -14 mV, and *ϕ*_h_ = 0.005.

All simulations in single compartment models were conducted in XPP. Bifurcation analysis was conducted using AUTO via the XPP interface. The multicompartment model was built in NEURON. Ion channels were modeled as above except that both *ḡ*_Na_ and *ḡ*_K_ were increased to 30 mS/cm^2^. Additional parameters were as follows: axial resistivity *R*_a_ = 150 Ω·cm, diameter = 1 μm, compartment length = 1 mm, *d_lambda* = 0.01. GABA conductance *ḡ*_GABA_ was modeled as a uniform density throughout the middle compartment.

### Dorsal root ganglion (DRG) neuron preparation and electrophysiology

All experiments were carried out on adult (200–340 g) male Sprague-Dawley rats (Harlan, Indianapolis, IN and Charles River, Montreal, Quebec). A subset of animals received spinal nerve ligation (SNL) 2–5 days before terminal experiments [74]. Under isoflurane anesthesia, the paraspinal muscles were separated to access the L6 process, which was carefully removed. The L5 spinal nerve was tightly ligated with 6–0 silk suture. All nerve-injured animals maintained motor function but developed neuropathic pain as inferred by guarding of the affected paw.

To collect DRG neurons, rats were deeply anesthetized by subcutaneous injection of anesthetic cocktail (1 ml/kg of 55 mg/ml ketamine, 5.5 mg/ml xylazine, and 1.1 mg/ml acepromazine) or by isoflurane (4% for induction; 2.5% for maintenance). DRG (L4 and L5 in naïve animals; L5 in nerve-injured animals) were surgically removed to chilled MEM-FBS culture media and desheathed. DRG were then enzymatically treated for 45 minutes in culture media composed of 89% MEM, 370 units/ml penicillin and 370 μg/ml streptomycin, 1% MEM vitamin solution (all from Life Technologies), and 1.2 mg/ml collagenase Type 4 (Worthington Biochemical Corp). DRG were mechanically dissociated by trituration with a fire-polished Pasteur pipette, and further enzymatically treated for 5 minutes in Ca^2+^- and Mg^2+^-free Hanks’ balanced salt solution (HBSS; Life Technologies Inc), containing 2.5 mg/ml trypsin (Worthington Biochemical Corp) and 0.02% sterile ethylenediaminetetraacetic acid (EDTA; Sigma-Aldrich Canada Ltd). Trypsin activity was subsequently inhibited by the addition of MEM-FBS supplemented with 0.625 mg/ml MgSO_4_ (Caledon Labs). Dissociated cells in MEM-FBS were plated on glass coverslips previously coated by a solution of 0.1 mg/ml poly-D-lysine, and incubated in MEM-FBS at 37°C, 5% CO_2_, and 90% humidity for 2 h. Coverslips were then transferred to a HEPES-buffered Leibovitz’s L-15 media containing glutamine (Life Technologies Ltd), 10% FBS, 100 units/ml of penicillin and 100 μg/ml streptomycin, and 5 mM D-glucose (Caledon Labs) and stored at room temperature until used for experiments for 2–28 hours later. Spiking properties do not change appreciably over this period and nor do neurites develop based on storage at room temperature, omission of laminin from coverslips, and the growth factor-free culture medium used.

Coverslips with cultured cells were transferred to a recording chamber constantly perfused with room temperature, oxygenated (95% O_2_ and 5% CO_2_) artificial cerebral spinal fluid containing (in mM) 126 NaCl, 2.5 KCl, 2 CaCl_2_, 2 MgCl_2_, 10 D-glucose, 26 NaHCO_3_, 1.25 NaH_2_PO_4_. Cells were recorded in the whole-cell configuration with >70% series resistance compensation using an Axopatch 200B amplifier (Molecular Devices; Palo Alto, CA). Electrodes (2–5 MΩ) were filled with a recording solution containing (in mM) 125 KMeSO_4_, 5 KCl, 10 HEPES, 2 MgCl_2_, 4 ATP, 0.4 GTP as well as 0.1% Lucifer Yellow; pH was adjusted to 7.2 with KOH and osmolality was between 270 and 290 mosmol/L. For experiments on the contribution of ANO-1 channels, KMeSO_4_ was reduced to 67 mM and KCl was increased to 63 mM to give E_Cl_ = -20 mV. Data were low-pass filtered at 2 KHz, digitized at 20 KHz using a CED 1401 computer interface (Cambridge Electronic Design, Cambridge, UK), and analyzed offline. Virtual GABA conductance was applied via dynamic clamp using Signal 5 software (CED). The virtual conductance was modeled as a step or as a synaptic waveform described by Eqn. 6. To express the virtual conductance as a density and thus exclude direct effects of cell size, we normalized absolute conductance values by membrane capacitance *C* because *C* is proportional to the surface area of the cell. Capacitance was measured for each cell based on responses to small (50 pA) hyperpolarizing current steps, where *C* = *τ*_membrane_ / *R*_in_. To increase cellular excitability in neurons from naïve animals, potassium channels were blocked with bath applied 4-aminopyridine (4-AP). In a subset of experiments with 4-AP, a virtual voltage-dependent sodium conductance was also inserted via dynamic clamp using the equations and parameters reported by Ratté et al. [45]. Neurons from nerve-injured animals are already hyperexcitable and were not, therefore, subject to manipulations (i.e. 4-AP or virtual sodium conductance) intended to increase excitability.

All data and computer code are available from the corresponding author upon request.

## Supporting Information

S1 Fig The neuron model used in Fig 1 was modified to reproduce the minimum *ḡ*_GABA_ required to evoke spiking observed experimentally in Fig 2. Specifically, we reduced the leak conductance *g*_leak_ to 0.7 mS/cm^2^ and increased the potassium conductance *ḡ*_K_ to 30 mS/cm^2^. All other parameters were unchanged. Testing with different *ḡ*_GABA_ (indicated on the figure), *E*_GABA_ and *β*_w_ were systematically varied like in Fig 1. Labels *a-d* are shown on top of graph shading, which depicts the distribution of excitability that could be expected within a heterogeneous population of neurons. Curves here show the minimum requirements for transient spiking. Note that for low values of *ḡ*_GABA_, a small increase in *ḡ*_GABA_ causes a large shift in the curves, whereas for high values of *ḡ*_GABA_, a large increase in *ḡ*_GABA_ causes little if any shift. Consistent with Fig 1, this re-affirms that increasing *ḡ*_GABA_ will not eventually cause spiking (depending on *E*_GABA_ and intrinsic neuronal excitability).
(TIF)
