# Supplementary figures and images for: Combined Changes in Chloride Regulation and Neuronal Excitability Enable Primary Afferent Depolarization to Elicit Spiking without Compromising its Inhibitory Effects
Source: PLoS Comput Biol. 2016 Nov 11;12(11):e1005215. doi: 10.1371/journal.pcbi.1005215 (PMC5105942; doi:10.1371/journal.pcbi.1005215)

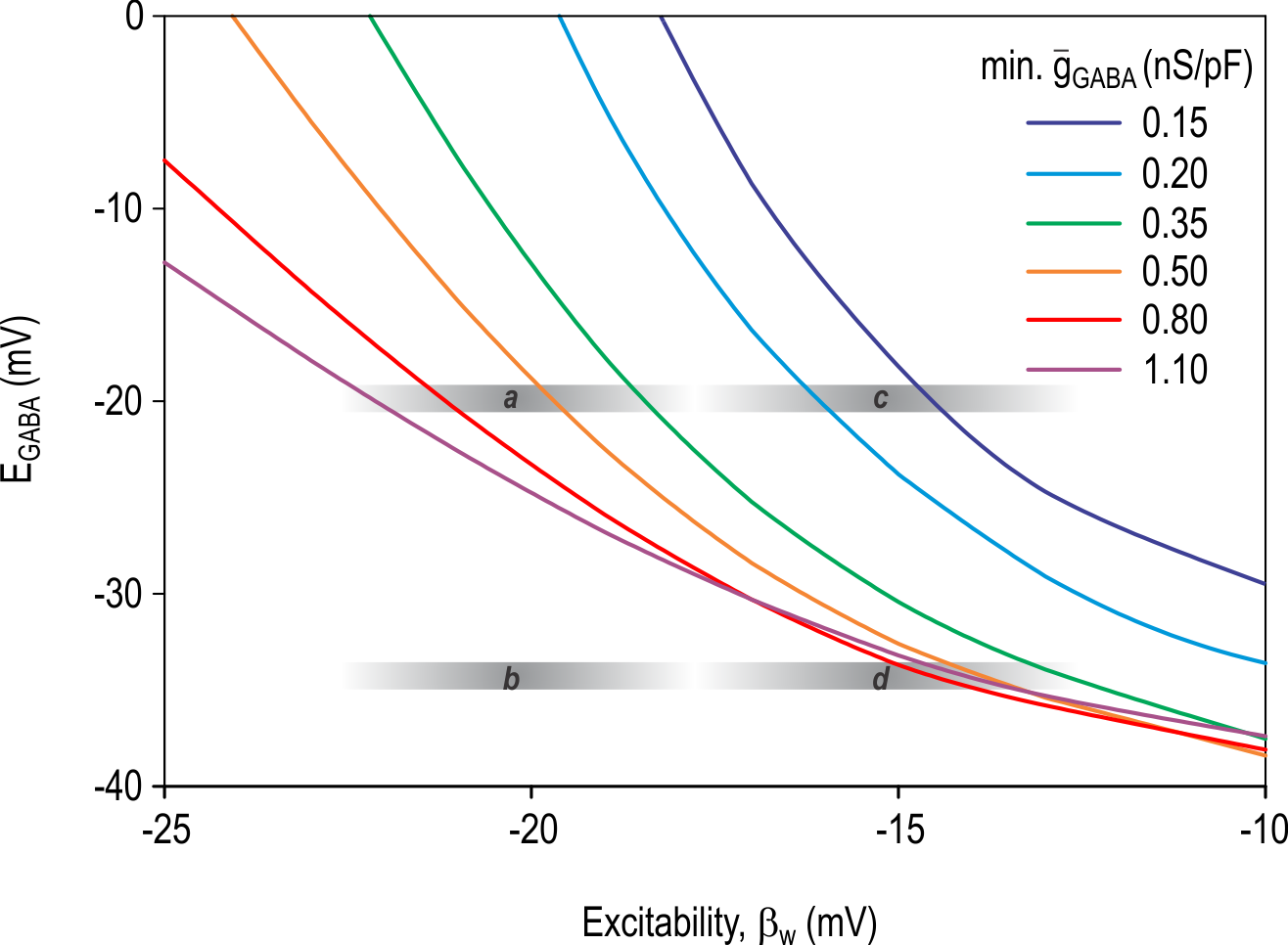

Supplement: S1 Fig — Specifically, we reduced the leak conductance gleak to 0.7 mS/cm2 and increased the potassium conductance ḡK to 30 mS/cm2. All other parameters were unchanged. Testing with different ḡGABA (indicated on the figure), EGABA and βw were systematically varied like in Fig 1. Labels a-d are shown on top of graph shading, which depicts the distribution of excitability that could be expected within a heterogeneous population of neurons. Curves here show the minimum requirements for transient spiking. Note that for low values of ḡGABA, a small increase in ḡGABA causes a large shift in the curves, whereas for high values of ḡGABA, a large increase in ḡGABA causes little if any shift. Consistent with Fig 1, this re-affirms that increasing ḡGABA will not eventually cause spiking (depending on EGABA and intrinsic neuronal excitability). (TIF) [file pcbi.1005215.s001.tif]
